# FcγRIIb blockage: a promising immunotherapy target for severe COVID-19

**DOI:** 10.1038/s41392-021-00590-8

**Published:** 2021-04-09

**Authors:** Xuemei He, Manni Wang, Min Wu

**Affiliations:** 1 Laboratory of Aging Research and Cancer Drug Target, State Key Laboratory of Biotherapy and Cancer Center, National Clinical Research Center for Geriatrics, West China Hospital, Sichuan University, Chengdu, Sichuan P. R. China; 2 Department of Biomedical Sciences, University of North Dakota School of Medicine and Health Sciences, Grand Forks, ND USA

**Keywords:** Infectious diseases, Infection

A recent study published in Nature by Combes et al.^1^ introduced a whole-blood-preserving single-cell analysis strategy to explore the contributions of the immune cells, including neutrophils, monocytes, platelets, and lymphocytes. The team comprehensively analyzed a variety of cellular and serological immune features between severe and mild to moderate phenotypes of patients with coronavirus disease 2019 (COVID-19), in order to identify potential targets of immunotherapies for the severe COVID-19 patients who need prompt and effective treatments.

The COVID-19 caused by severe acute respiratory syndrome-coronavirus 2 (SARS-CoV-2) has become a life-threatening global problem since its outbreak in January 2020. As of February 14, 2021, over 108 million people have been infected with SARS-CoV-2 worldwide. Although majority of patients have no symptoms or mild to moderate disease, around 14% of patients exhibit severe disease and 5% of patients show most severe disease with high mortality risk. In severe disease cases, patients may develop serious pneumonia or acute respiratory distress syndrome (ARDS) with 40–50% death rates.^2^ Therefore, there is an urgent need for a comprehensive understanding of the differences between severe and mild phenotypes of COVID-19, which might enable us to identify potential therapeutic targets and develop highly effective strategies for coping with severe COVID-19.

**Fig. 1:**
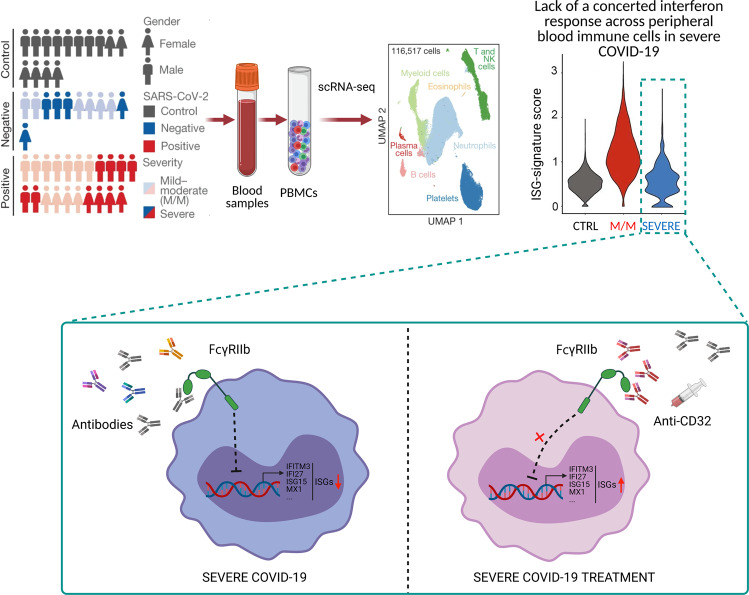
Immune phenotyping of red-blood-cell-depleted blood samples but preserving all white blood cells and other serum components of patients with COVID19 using scRNA-seq analysis. Antibodies from patients with severe COVID-19 neutralized ISG induction by binding of FcγRIIb to contribute to the damaging effects in the suffered. Blocking the antibodies targeting FcγRIIb restored the induction of ISGs in PBMCs to reduce the severity of disease in dying patients, ushering in an advancement of immune regulation in the most severe disease and indicating potential effective therapeutic approaches

Previous studies have reported that severe COVID-19 was associated with peripheral immune activities, including increased inflammatory monocytes, lymphopenia and T cell exhaustion.^3^ Wilk et al.^3^ have elucidated a unique peripheral immune cell phenotype in COVID-19, including a heterogeneous interferon-stimulated gene (ISG) signature. Inspired by these encouraging results, Combes et al. performed a comprehensive patient blood cell analysis and revealed the role of ISG signature in the peripheral blood immunocytes of patients with mild-moderate or severe COVID-19 diseases. They enrolled 14 healthy control individuals, 21 in-patients positive for SARS-CoV-2 including 11 patients with mild-moderate COVID-19 and 10 patients with severe COVID-19, and 11 in-patients with similar clinical presentations caused by other infections or unknown origin but negative for SARS-CoV-2 as acute lung injury controls (Fig. 1). Patients classified as mild-moderate remained mild-moderate during hospitalization, suggesting that mild-moderate and severe state are at stable stages (rather than transient phases) of disease. Analyses of this study suggest that the intrinsic immune systems residing in different individuals contribute enormously to the disease pathogenesis and progression of COVID-19. One notable feature was observed in the scRNA-seq assessment that neutrophil penetration was positively correlated with disease severity, whereas lymphocyte populations were inversely related to disease severity in both SARS-CoV-2 positive or negative individuals. Further, an ISG-signature neutrophil population was highly enriched in mild-moderate patients, specifically within the individuals who were with mild-moderate COVID-19 rather than severe disease. Akin to neutrophils, ISG-signature scores in monocytes, lymphoid cells, and platelets from patients with mild-moderate disease were also elevated but were significantly decreased in those from patients with severe COVID-19, strongly indicating an inverse correlation between ISG^+^ cell populations and disease severity in the clinic (Fig. 1).

It has been long considered that viral load is associated with severity and mortality in COVID-19. Paradoxically, the authors discovered that patients with severe COVID-19 had higher levels of neutralizing antibodies and a lower viral load than mild-moderate COVID-19 patients. This contradiction might be explained by the previous studies that examined patients with high mortality but few such patients in this cohort. Notably, the severe COVID-19 patients maintained higher antibody titers compared to patients with mild-moderate disease over time. Furthermore, only 1 of 21 patients with mild-moderate disease developed into severe disease without correlation of ISG^+^ cell populations presence. These results argue against a simple temporal relationship between mild-moderate and severe diseases, and indicate an etiology for this descrepancy in states in the serum. Nevertheless, the current study is based on a small population cohort from a single clinic and may not represent all populations of COVID-19 patients in general, which warrant further thorough investigation.

An obvious paradox was observed in patients with severe COVID-19 that some individuals had high levels of serum interferon α (IFNα) and potentially neutralizing antibodies but low ISG-signature scores, indicating that the sera from patients with severe COVID-19 might contain certain substances that could suppress the IFN response. Since antibodies against IFNα were present in a minority of patients with COVID-19, which is consistent with a previous study that anti-IFNα antibodies were present in approximately 12% of patients with COVID-19,^4^ IFNα reactivity alone may not explain the lack of ISG^+^ cells in the majority of patients with severe COVID-19. Interestingly, the serum of severe COVID-19 patients showed from partial inhibition to complete block of the IFNα response, and depletion of the antibodies in serum of severe COVID-19 patient with protein A and protein G beads restored the IFNα-dependent generation of the ISG signature in monocytes and lymphocytes. Remarkably, blocking antibodies towards the Fc receptors (CD16, CD64, and CD32) restored the induction of ISGs, including IFITM3, IFI27, ISG15, and MX1, indicating that antibodies in the sera of patients with severe disease trigger Fc receptor signaling to restrain transcriptional responses (Fig. 1). It should be noted that earlier studies demonstrate that impeding the Fc γ receptor IIb (FcγRIIb, also known as CD32B) blockade caused higher levels of ISGs in dendritic cells and monocytes, whereas inhibiting FcγRIIa altered the outcome of anti-viral response.^5^ Consistent with this observation, Combes et al.^1^ found that blocking FcγRIIb rather than blocking antibodies to CD16 or CD64 rescued the induction of ISGs in monocytes cultured with the sera from patients with severe COVID-19.

Next, the authors delved into analysis of the specific natures of targeted proteins and/or other molecules by the auto-antibodies from the COVID-19 patients, and found that these antibodies can bind to numerous targets in the cells including phospholipids and endothelial proteins. However, antibodies from different patients may show distinct and diverse binding specificities rather than universal patterns across all patients. This important discovery represents a major insight into the separate, distinct mechanism in the regulation of host homeostasis, inflammation, and direct viral killing because based on the clinical data, the increased anti-viral antibodies in severe disease cases could not protect against SARS-CoV-2 infection, rather aggravating the progression of disease. This insight about targeting the inhibited negative regulation facilitated by FcγRIIb can serve as a basis to further develop more effective drugs and reagents to tackle the specific issues with severe COVID-19.

Taken together, the study by Combes and colleagues highlights the coordinated pattern of induced ISGs expression in blood immune cells as a significant distinction between severe and mild-moderate phenotypes of COVID-19, and emphasizes that the antibodies in the sera of severe COVID-19 patients neutralized ISG induction by impacting specific Fc receptors signaling instead of targeting the entire ISG group. Moreover, the authors suggest potential strategies, such as rituximab, antibodies specific against FcγRIIb, might be able to enhance the IFN response and restore the weakened antiviral immune responses in patients with severe COVID-19. It is highly challenging to design drugs or reagents to specifically remove these antibodies that bind to FcγRIIb receptors in people with severe COVID-19, because antibodies may often exhibit cross-reaction with other molecules of similar epitopes.

Further studies are warranted to characterize the specificity and applicability of these antibodies in treating COVID-19, and other major infectious diseases as Combes and colleagues used a small population cohort from a single clinic may only represent limited patient coverage in each group. Critically, they did not use a general ratio of severe and mild–moderate phenotypes of patients (15% vs. 80%) to represent the data profile in general populations. Nevertheless, it is appealing to apply this type of global analysis with human samples to identify other highly relevant targets in COVID-19 and beyond. Overall, the study ushers in a technical merit in finding potential treatments for COVID-19 patients through a global screening analysis like this whole-blood-preserving single-cell study, with a much large population of patient at the international level. The further development and application of FcγRIIb blockage may also offer an immediate, promising therapeutic strategy for severe COVID-19.
